# Realist Case Study Research: A Methodological Guide

**DOI:** 10.5334/pme.3269

**Published:** 2026-11-03

**Authors:** Anél Wiese, Deirdre Bennett

**Affiliations:** 1 Medical Education Unit, School of Medicine, University College Cork, Cork, Ireland

## Abstract

Much of what happens in health professions education (HPE) is intervention work in complex systems, and researchers now expect methodologies that go beyond whether a programme worked to explain how and why it worked and for whom. Realist evaluation and case study research each speak to that need, and they have developed alongside one another. Their purposeful integration, however, has not been articulated as a methodology in its own right. We draw on realist evaluation theory and on case study methodology informed by our own realist programme in supervised workplace learning, to describe the integration. We call it realist case study research. What follows describes what makes the pairing more than the sum of its parts and works through the phases of the approach using a single running study. We discuss where the approach is useful and where it is not.

Realist case study research uses realist evaluation logic, expressed through programme theory and context-mechanism-outcome (CMO) configurations, to drive bounded case inquiry. Case study methodology gives the realist project the disciplined boundaries and thick within-case description it needs. Realist logic gives case study research an explanatory spine and a systematic way of comparing across cases that moves past thematic reading. The result is a refined programme theory, tested against the messy particulars of practice and portable to new settings.

## Introduction

Much of what happens in health professions education (HPE) involves complex interventions operating in complex systems. Workplace learning, simulation programmes, curricula, and faculty development all sit inside settings that shape whether they work and for whom. However, this is not the whole of HPE research. A substantial body of scholarship in this field studies phenomena that are not helpfully framed as interventions at all, from professional identity formation to validity as an argument. Our paper speaks to the subset of HPE research questions that ask how a designed intervention behaves in real contexts.

Realist evaluation addresses these questions. First set out by Pawson and Tilley [1], realist evaluation has gained real traction in HPE [2]. Its central claim is that outcomes (O) are produced by mechanisms (M) that are triggered (or not) by contexts (C). The realist family of methods has grown alongside it, with realist reviews [3], realist evaluation [1,4], realist interviewing [5], and realist analysis [6] each now articulated as distinct methods. Case study research has travelled in parallel. Drawing on the foundational work of Yin [7], Stake [8], and Merriam [9], it offers bounded inquiry, contextual depth, multiple data sources, and an ability to hold a phenomenon together as a situated whole. It is well established in HPE [10] and has had renewed attention through Greenhalgh’s taxonomy of case study approaches [11].

The two traditions meet in practice more often than the literature reflects. HPE lacks an explicit methodological guide to realist case study research, despite scattered examples in neighbouring fields. There are papers combining critical realism, in Bhaskar’s tradition [12], with case study methodology, and these draw on a different philosophical lineage from Pawson’s realist evaluation [13,14,15]. There are realist evaluations that use case as a research design without articulating the integration [16,17,18]. And Greenhalgh’s case study taxonomy recognises cases informed by realist evaluation as a category, but does not elaborate on the methodology [11]. The result is that researchers who want to conduct realist case study research in HPE have no unified statement to work from.

We aim to fill that gap. We articulate realist case study research as a purposefully integrated methodological approach and offer practical guidance drawn from our own realist programme on supervised workplace learning in postgraduate medical training [19,20]. That running study threads through the phase-by-phase account later in the paper.

## What is realist case study research?

Realist case study research is a methodology that uses realist evaluation logic to drive case study inquiry. Programme theory and CMO configurations set the analytical agenda, with generative causation as the underlying causal claim. Case selection carries that agenda into the field, and within-case and cross-case analysis carry it through to a refined theory. This integration gives cases a defined analytical role beyond serving as sites for data collection.

The philosophical footing sits in scientific realism as articulated by Pawson [21]. Causation is generative rather than successionist, so programmes work or fail because they introduce resources or opportunities that are taken up differently by different people in different circumstances. Context is not a nuisance variable to be controlled but a fundamental explanatory category. Realist case study research inherits its ontological and epistemological commitments from scientific realism rather than from case study research itself. That inheritance matters, because case study research does not belong to a single paradigm. Yin’s tradition is broadly post-positivist [7]. Stake’s tradition is interpretivist [8]. Merriam is more pragmatic [9]. Case study is a methodological container whose commitments are supplied by the research programme within which it is deployed. Once it enters the realist project, the paradigm becomes scientific realism, with a mind-independent but stratified reality and an explanatory rather than descriptive ambition.

We see a close fit between realist case study research and Yin’s theory-informed approach to case study. Yin uses theoretical propositions to guide inquiry and replication logic to select cases expected to produce similar findings or contrasting findings for theoretically anticipated reasons [7]. Realist evaluation likewise develops and tests programme theories, asking how mechanisms generate outcomes under particular contextual conditions [1]. These approaches provide compatible grounds for selecting cases according to their contribution to explanation. Their concepts should nevertheless remain distinct. Demi-regularities are recurring, semi-predictable patterns that inform realist theory refinement [6], whereas analytic generalisation concerns extending case findings to theoretical propositions beyond the cases studied [22]. We therefore locate the alignment in their shared commitment to testing and refining explanations through theoretically informed inquiry, without treating their analytical procedures as interchangeable [1,7].

The CMO configuration is the signature heuristic of this approach. Dalkin and colleagues clarified that mechanisms comprise both the resources offered by an intervention and the reasoning or responses of participants [23], building on earlier work unpacking mechanisms [24]. Within realist case study research, CMO configurations serve as the unit of analysis. The researcher identifies, within each case, how contextual features triggered or inhibited particular mechanisms and produced observed outcomes.

## The purpose of the case

The case is a theoretical instrument. While case study research provides a methodological container for different philosophical commitments, the case itself is the bounded object of inquiry through which we test and refine programme theory. Its purpose extends beyond holding data or providing a description of a setting. The case earns its place by offering a theoretically defended chance to see whether, and how, a set of CMO conjectures holds up. Case selection therefore weighs theoretical relevance alongside practical access, while within-case analysis preserves the circumstances in which the explanation takes shape.

## Theory-data dialogue

Realist case study research involves a tension between theoretical guidance and openness to the case. Realist research is theory-first. The programme theory sets the questions and the analytical apparatus, and it determines the terms of case selection. Case study research in Stake’s tradition, by contrast, has a strong instinct to let the case speak for itself, with theory arriving later, from the data [8]. The instinct behind that objection is a good one, because a case that cannot push back on the theory is not doing the work a case is meant to do.

We take the position that this tension is productive rather than fatal. Theory sets the initial frame and the analytical vocabulary, and the case data can and should push back on it. Anomalies and unexpected couplings are not failures of the programme theory. They are the material from which programme theory refinement is built. This dialogue between theory and data is the engine of the approach. It is also why the case boundary is defended before fieldwork but revisable during it. The researcher who enters the field committed to protecting the initial theory from disconfirmation is not conducting realist case study research.

## From within-case explanation to cross-case comparison

The integration becomes most visible in the move from a case-level explanation to comparison across cases. Theory-informed case selection establishes why particular cases should be compared [7]. Within each case, the researcher then examines how the proposed explanation holds up against the evidence, retaining enough contextual detail to account for unexpected findings. A programme, an organisation, a training site, or a learner’s trajectory can provide the boundary for that inquiry [8,25]. The boundary keeps the explanation connected to the circumstances in which it was developed [26].

CMO configurations provide a common basis for comparing these case-level explanations [27]. The question is whether a proposed mechanism helps explain the outcomes in each case and which contextual conditions enable or constrain its operation. Recurring patterns can support candidate demi-regularities, while contrasts help identify conditions under which the explanation needs to change [21,28]. This comparison does not assume that cases with different contexts have identical CMO configurations. It tests the explanatory relationship proposed across those differences.

The case-level account remains part of the evidence throughout this comparison. Retaining it helps researchers avoid combining fragments from different settings into a configuration that no individual case supports. The resulting programme theory can reach beyond the immediate cases, but its scope remains bounded by the variation examined and the evidence available. In a single-case design, comparison within the case or over time can serve a similar explanatory purpose without becoming cross-case analysis.

## Distinguishing realist case study research from adjacent designs

The distinction from a single-site realist evaluation rests on the role of the case, rather than on the number of settings. A realist evaluation can also use theoretically defined cases and detailed within-case accounts. In the approach described here, we make those commitments explicit. The researcher enters the field with a theoretically defended case boundary and revisits it as fieldwork reveals what the case contains in practice. Each case is reconstructed as a situated whole with its own contextual narrative and case-level CMO account before cross-case comparison begins. Multiple sources of evidence can deepen that account, but their selection should follow the explanatory question rather than a fixed minimum number of data types [7,8]. Revisions to boundaries or evidence sources should be justified and documented. Table 1 sets out these commitments alongside single-site and multi-site realist evaluations. It also includes critical realist case study, an adjacent approach that need not organise its explanation around programme theory and CMO configurations.

**Table 1:** Distinguishing Realist Case Study Research from Adjacent Realist and Case Study Designs.


FEATURE	REALIST CASE STUDY RESEARCH	SINGLE-SITE REALIST EVALUATION	MULTI-SITE REALIST EVALUATION	CRITICAL REALIST CASE STUDY

Theoretical bounding of cases	Required; theoretically justified and revisable during fieldwork	Optional; site is often pragmatically chosen	Optional; sites may be pragmatically chosen	Required; case defined by Bhaskarian ontology

Within-case narrative integrity	Required; each case reconstructed as a situated whole before cross-case work	Variable; data often pooled across the site	Often weak; data pooled across sites for CMO building	Strong; case as stratified whole

Multiple data sources	Evidence selected for explanatory depth; multiple sources where appropriate	Optional; often interview-dominant	Optional; often interview-dominant	Typical

Programme theory as analytical spine	Required; programme theory guides analysis within bounded cases	Required	Required	Optional; retroduction is the analytical strategy

Cross-case comparison	For multiple-case designs; compare explanations across cases	Not applicable	Required for demi-regularities	Optional

Philosophical foundation	Scientific realism (Pawson)	Scientific realism (Pawson)	Scientific realism (Pawson)	Critical realism (Bhaskar)

Primary product	Refined, transferable programme theory grounded in case-level evidence	Refined programme theory for the site	Refined programme theory across sites	Explanatory account of generative mechanisms


## Conducting realist case study research

The six phases below carry realist case study research from an initial programme theory to a refined one. We present them sequentially for clarity, but in practice the work is iterative. Table 2 summarises the key decisions researchers face at each phase. To make the phases concrete, we thread through our own study of supervised workplace learning in postgraduate medical training as a running illustration [19,20]. That study asked how supervision shapes residents’ learning across training sites. Box 1 provides a teaching reconstruction of the analytical steps using findings from that study.

**Table 2:** Practical Decision Points in Realist Case Study Research.


PHASE	KEY DECISION	CONSIDERATIONS	GUIDANCE

**Developing the IPT**	How detailed should the initial programme theory be?	Overly detailed theories may constrain inquiry. Overly vague theories provide insufficient direction.	Express CMOs at a level of specificity that guides case selection and data collection. Expect refinement.

**Developing the IPT**	What sources should inform the IPT?	Realist review, literature, stakeholder consultation, programme documents	A prior realist review is ideal but not essential. Use multiple sources to triangulate the initial theory.

**Selecting cases**	Single or multiple cases?	Single cases offer depth. Multiple cases enable cross-case CMO comparison.	Multiple cases are preferred when the aim is to compare how CMOs operate across contexts. Ensure depth is maintained.

**Selecting cases**	How many cases?	Practical constraints of time, access, and analytical capacity	Typically 2–6 cases. Enough for meaningful comparison without sacrificing within-case depth.

**Selecting cases**	What defines the case boundary?	Must be clear and theoretically justified	Boundaries should align with the programme theory. Specify temporal, spatial, and conceptual limits.

**Data collection**	Which data sources within each case?	Multiple sources can deepen contextual understanding and test explanations.	Select sources that address the programme theory. Justify the choice and acknowledge evidence gaps.

**Data collection**	How to handle iteration?	Emerging findings should shape subsequent data collection.	Build in flexibility. Schedule data collection in waves where possible. Maintain an audit trail.

**Within-case analysis**	How to move from codes to CMO configurations?	Avoid fragmenting data into decontextualised themes.	Code with CMO logic from the start. Map data against the programme theory. Construct case-level explanatory accounts.

**Cross-case analysis**	How to compare CMOs systematically?	Need a consistent framework for comparison	Use a matrix or tabular format. Compare like-for-like CMOs across cases. Attend to both patterns and exceptions.

**Theory refinement**	When is the programme theory sufficiently refined?	Diminishing new insights from additional cases or data	When the theory accounts for observed outcomes across cases with coherent CMO explanations. Acknowledge remaining uncertainties.

**Box 1 A worked example. From within-case evidence to cross-case explanation**

This teaching reconstruction draws on the published synthesis and case-study findings. It illustrates the analytical steps rather than reproducing the original coding sequence [19,20].

**Initial theoretical basis**

The synthesis identified feedback as a mechanism within dialogue during practice and entrustment within supervised participation. These mechanisms informed the subsequent case study [19,20].

**Published interview excerpt**

A junior resident in surgery described feedback during procedures. “In theatre…if you were doing something, there will always be feedback…how you could improve it or what you were doing well” (JR6, C2) [20].

**Illustrative within-case configuration**

In surgery, directly observable procedural work and scarce theatre access (C) shaped performance-contingent entrustment coupled with monitoring and feedback (M), producing staged participation for engaged residents (O). Progression was uneven because access also depended on signalling engagement [20].

**Cross-case refinement**

Geriatrics also involved close monitoring and frequent feedback, but these coupled with narrow entrustment and limited progression in autonomy. The contrast shifts the explanation from the presence of feedback to how feedback and entrustment interact within local conditions. These are context-dependent patterns, not fixed specialty characteristics [20].

### Phase 1. Developing the initial programme theory

Every realist case study begins with an initial programme theory (IPT), a set of conjectured CMO configurations that describe how the intervention is expected to work, for whom, and in what circumstances [1]. The IPT is not a hypothesis to be confirmed or refuted, but a theoretical framework to be tested and refined through empirical inquiry. RAMESES II emphasises articulating programme theory explicitly and transparently from the outset [4]. Sources include prior realist reviews, existing empirical literature, programme documents, policy frameworks, and stakeholder consultation. The last of these can be structured through realist diagnostic workshops to elicit and refine programme theory with those closest to the intervention [29]. The IPT should be expressed with enough specificity to guide case selection and data collection, but not so tightly that it forecloses what the field can reveal.

In our study, the IPT came from a prior realist synthesis of supervised workplace learning. That synthesis identified entrustment and support seeking within supervised participation in practice. It also identified monitoring and modelling in observation of practice, with meaning making and feedback informing dialogue during practice [19]. We used these mechanisms as sensitising concepts when examining supervision within and across the cases, allowing the empirical analysis to extend the theoretical account [20].

### Phase 2. Defining and selecting cases

Cases are selected purposively to test and refine the IPT. The selection criteria come directly from the programme theory. The researcher chooses cases that represent different contexts predicted to trigger, or fail to trigger, the theorised mechanisms [1]. If the IPT proposes that a supportive supervisory culture (context) enables help-seeking behaviour (mechanism), then cases should include settings with strong and weak supervisory cultures, so the mechanism can be tested where it is expected to fire and where it is not.

The decision between single and multiple cases depends on the research question. Single cases are appropriate when a case offers a critical opportunity to test the IPT [7]. Multiple cases are useful in realist case study research because they enable comparison of CMO configurations across different contexts, which is where the approach makes its distinctive analytical contribution. However, depth must not be sacrificed for breadth, so the researcher must be able to conduct in-depth inquiry within each case.

There is no numeric rule for case number. Sufficiency is reached when additional cases no longer yield new CMO configurations or substantially challenge the programme theory. In our experience, two to six cases are typical for HPE-scale projects, but this is a function of the programme theory’s complexity and the variation across feasible sites, not a quota. Ideal theoretical variation is often not achievable, because researchers work with the cases they can access. If the theory implies that a mechanism will behave differently in a low-resource rural setting than in a well-resourced teaching hospital and only teaching hospitals will grant access, then the study can only test the theory across the range it could sample. The demi-regularities that emerge are bounded by that range, and this should be stated.

In our own study, we selected four clinical departments within the Irish healthcare system [20]. The macro context of national training regulation was shared. The meso context, the culture and organisation of supervision in each department, varied substantially, and this variation was the analytic focus. The cases provided variation within one national system rather than representing the full range of postgraduate training settings [20].

### Phase 3. Data collection within cases

Data collection within each case is guided by the programme theory. Multiple data sources can deepen contextual understanding, while the choice of evidence depends on the question and what can be accessed. Realist interviews can combine open questions about experience with theory-informed probes, allowing participants to challenge the researcher’s explanation [5,30]. Data collection can proceed iteratively, with early findings informing later questions. Researchers should explain how their evidence supports the case-level account and where gaps remain.

In our study of supervised workplace learning, we used semi-structured interviews with residents and attending physicians across four clinical departments. Participants described everyday supervisory relationships and learning opportunities through concrete examples. We did not supply predefined definitions of supervision or mechanisms. This allowed us to relate participants’ accounts to the existing realist theory without requiring them to adopt its vocabulary [20]. The interviews captured accounts of supervision rather than direct observations of practice, which bounded what we could infer from them [20].

### Phase 4. Within-case analysis

For each case, the researcher identifies the CMO configurations that operated. This goes beyond thematic coding because the analytical task is configurational and seeks to explain the pattern of outcomes observed within the case by tracing how context triggered, or failed to trigger, specific mechanisms [31]. Within-case analysis involves mapping the data against the IPT and asking which theorised CMOs are supported by the evidence of this case, and which are contradicted or displaced by unexpected configurations. The RAMESES II standards emphasise making these analytical moves transparent [4]. The aim is a case-level account that explains why this case produced its particular pattern of outcomes.

Within-case analysis in each of our four departments produced a situated account of supervision. Examining each case as a whole showed how mechanisms such as entrustment and feedback operated in relation to one another [20]. Box 1 reconstructs an illustrative sequence from the published findings to show how a case-level account can inform cross-case explanation.

### Phase 5. Cross-case analysis

Cross-case analysis is where realist case study research makes its most distinctive contribution. The researcher compares CMO configurations across cases, using systematic techniques such as matrix displays to identify patterns [7]. The analytical questions concern which recurring patterns suggest candidate demi-regularities and which contextual differences explain variation in outcomes.

Attending to the layered nature of context helps to sharpen the comparison. Context operates at more than one level at the same time. Micro-level contexts include specific supervisor-resident relationships or local team dynamics. Meso-level context concerns organisational features, such as a training site’s culture. Macro-level context concerns system-level features, such as regulatory frameworks or professional norms. Multiple-case designs may hold macro context relatively constant and vary meso context systematically through case selection. This lets researchers see how mechanisms at the micro level are enabled or constrained by variation at the meso or micro level, and identify which CMO configurations are stable across the macro context and which are meso- or micro-context-dependent.

In our study, differences between departments shaped how supervisory mechanisms operated together. Geriatric medicine and surgery both involved close monitoring and frequent feedback, yet their configurations supported different learning experiences. In geriatrics, monitoring reinforced narrow entrustment and limited progression in autonomy. In surgery, monitoring informed performance-contingent entrustment and feedback shaped subsequent opportunities to participate. The cross-case comparison therefore refined the explanation towards how mechanisms coupled under local conditions, rather than whether individual mechanisms were present [20].

This configurational approach differs fundamentally from cross-case thematic analysis. Rather than asking what themes recur, the researcher asks what configurations recur and what explains the variation. This preserves the explanatory logic that is central to the realist enterprise and prevents the fragmentation that occurs when context and mechanism data are separated from outcomes in the analytical process.

### Phase 6. Refining programme theory

The end product of realist case study research is a refined programme theory of empirically tested and revised CMO configurations that explain how, why, for whom, and in what circumstances the intervention works or fails to work [1]. RAMESES II gives guidance on reporting refined programme theory transparently [4]. The refined theory is not a set of context-bound findings applicable only to the cases studied. It is a transferable explanatory framework that can be tested in new contexts, and its value lies in that transferability. Our published account of supervised workplace learning illustrates this end product [20]. The refined programme theory articulates how mechanisms of supervised workplace learning couple to shape residents’ engagement with supervised work.

## Quality in realist case study research

Quality in realist case study research has more than one source. It requires an explicit IPT and plausible CMO reasoning grounded in data. Researchers must also show how the programme theory changed, as emphasised in RAMESES II [4]. Case study research adds the need for justified case boundaries and detailed within-case accounts. The selection and integration of evidence must support those accounts [7,8].

The integration itself asks for a further test. Did the case serve as a test of theory, or was it used as an illustration of a theory already held to be true? Is the theory refinement grounded in case-level evidence, or has the researcher generalised across cases without doing the within-case work first? Reviewers should be able to trace, for any refined CMO, the case-level evidence that supported it and the evidence that pushed the original conjecture in that direction.

## Strengths and challenges

Realist case study research produces explanations rather than descriptions. By combining the depth of case study inquiry with the explanatory logic of CMO configurations, it generates accounts that explain why interventions behave differently across contexts. The refined programme theories it produces are transferable because they are expressed as CMO configurations that can be tested in new settings, and this makes them useful for cumulative knowledge building across HPE. The systematic approach to cross-case comparison, in which the researcher compares configurations rather than themes, provides analytical rigour that thematic multi-case designs do not offer.

The approach is also demanding. It requires prior theoretical work, ideally a realist review, though other sources can serve. Managing CMO configurations within and across cases asks for methodological competence in both realist evaluation and case study methodology, a dual expertise that may require targeted training or team collaboration. It is also more time-intensive and resource-intensive than shorter, single-method designs.

It is most appropriate when the research question asks how and why a complex intervention works differently across contexts, when existing programme theory is available to test, and when bounded settings need to be understood as wholes before they are compared. It is not the right choice when the question is purely descriptive, when there is no prior theory to work with, when context is not expected to matter, or when resources do not allow the in-depth case engagement that the methodology demands. Standard realist evaluation, standard case study research, or another qualitative approach will do better in those cases.

## Discussion

We set out to articulate realist case study research as a purposefully integrated methodological approach for HPE. Our argument has been that the integration produces methodological synergy, and together the two traditions do what neither does alone. We have described one coherent way of doing realist case study research, grounded in Pawson’s tradition. Other variants, more explicitly critical realist or complexity-informed, are equally legitimate developments of the integration.

Realist case study research fills a real gap in the HPE methodological toolkit, and it complements rather than replaces existing approaches. The growing family of realist methods in HPE has been articulated mostly at the level of reviews or as techniques within broader evaluations. Realist case study research adds a primary research approach that combines the explanatory ambition of the realist tradition with the in-depth, bounded inquiry of case study methodology. It suits questions HPE researchers frequently ask, from implementation research and curriculum evaluation through faculty development to workplace learning.

The CMO heuristic also has limits. In systems with strong feedback loops or temporally extended causation, simple C + M = O configurations risk under-representing what is going on. Researchers may need to examine coupled mechanisms and trace how configurations change over time. Realist case study research can reveal these complications through its within-case depth. Our study of supervised workplace learning offers a concrete example. Feedback in geriatrics was closely linked to correction and attending physician oversight, with limited progression in resident autonomy. In surgery, feedback coupled with observed performance and staged entrustment to shape future opportunities for participation. The explanatory difference lay in how feedback functioned within each configuration, not simply in its presence [20]. Representing those relationships requires an account of how mechanisms reinforce or constrain one another.

We acknowledge that this articulation is shaped by our own programme of realist research on supervised workplace learning in postgraduate training [19,20]. The methodological emphases described here, sustained engagement with each of a small number of theoretically defended sites and residents’ and supervisors’ perspectives within each case, reflect what was feasible and productive in that programme. Researchers working in different contexts, for example primary care or doctoral projects with constrained access, will need to adapt these emphases. The integrated approach we describe is principled rather than prescriptive.

Directions for future development follow from what we have described. Reporting standards specific to realist case study research would support both methodological rigour and the transparency needed to assess published studies. The RAMESES II standards [4] are an important starting point, and the protocol paper sets out the framework [32], but the case study dimension asks for additional considerations, from justification of case boundaries to the relationship between case-level findings and cross-case synthesis, and an explicit audit trail of programme theory refinement. Further methodological work is also needed on analytical techniques for managing coupled mechanisms across multiple cases. Methodological pluralism, not paradigmatic allegiance, should guide the choices researchers make [33].

This paper offers a starting point. The approach will evolve as more researchers use and adapt it, and we welcome dialogue about its application and limitations. We hope to have shown why realist case study research deserves a place in HPE researchers’ methodological repertoire as a coherent and practically useful approach.
